# PD-BertEDL: An Ensemble Deep Learning Method Using BERT and Multivariate Representation to Predict Peptide Detectability

**DOI:** 10.3390/ijms232012385

**Published:** 2022-10-16

**Authors:** Huiqing Wang, Juan Wang, Zhipeng Feng, Ying Li, Hong Zhao

**Affiliations:** College of Information and Computer, Taiyuan University of Technology, Taiyuan 030024, China

**Keywords:** peptide detectability, BERT, multivariate representation, ensemble deep learning

## Abstract

Peptide detectability is defined as the probability of identifying a peptide from a mixture of standard samples, which is a key step in protein identification and analysis. Exploring effective methods for predicting peptide detectability is helpful for disease treatment and clinical research. However, most existing computational methods for predicting peptide detectability rely on a single information. With the increasing complexity of feature representation, it is necessary to explore the influence of multivariate information on peptide detectability. Thus, we propose an ensemble deep learning method, PD-BertEDL. Bidirectional encoder representations from transformers (BERT) is introduced to capture the context information of peptides. Context information, sequence information, and physicochemical information of peptides were combined to construct the multivariate feature space of peptides. We use different deep learning methods to capture the high-quality features of different categories of peptides information and use the average fusion strategy to integrate three model prediction results to solve the heterogeneity problem and to enhance the robustness and adaptability of the model. The experimental results show that PD-BertEDL is superior to the existing prediction methods, which can effectively predict peptide detectability and provide strong support for protein identification and quantitative analysis, as well as disease treatment.

## 1. Introduction

Peptide detectability is defined as the probability of detecting a peptide from a standard sample analyzed by a proteomics program [1]. It is used to measure the relationship between the amount of protein in the sample and the peptide detected. In a shotgun protein assay, proteins in the mixture sample are enzymatically decomposed into peptides, and high-throughput peptide analysis is performed by liquid chromatography-tandem mass spectrometry (LC-MS/MS) and other techniques to determine the composition and content of proteins in the sample [1,2,3]. In this process, non-site cleavage of sequences, loss of peptide generated during enzymatic hydrolysis, and other abnormalities may lead to the deviation of the probability of peptide detection [2], thus restricting the identification and quantitative calculation of proteins. Studies have shown that peptide detectability is crucial to detection, analysis, and differential expression of proteins in proteomics [4,5,6]. Therefore, accurate prediction of peptide detectability helps protein detection and expression analysis provides reference value for the discovery of disease biomarkers and clinical research and thus helps us to more deeply understand cell biology and the underlying mechanisms of human diseases.

There is a strong correlation between amino acids and amino acids in the sequence. Researchers introduce contextual information as the feature representation of the peptide sequence level to better describe the global information of the sequence. Charoenkwan et al. [7] captured the context information of bitter peptides and used it as a feature, and used BiLSTM and DNN to identify bitter peptides. The results showed that the context information could effectively improve the recognition accuracy of bitter peptides. In peptide detectability, Serrano et al. [8] regarded amino acids as words, utilized word2vec to calculate the embedding vector of each word, and input the convolutional neural network to predict peptide detectability. Cheng et al. [9] used transformer and bidirectional gated neural network (BiGRU) to capture the context information of peptides, and improved the prediction performance of peptide detectability. These methods capture the context information of peptides and reflect the global information of peptides better. However, word2vec regards amino acids as words to calculate the embedding vector of each amino acid, but the vector is a fixed value. For the same amino acid, the embedding vector is the same regardless of its context, so it cannot fully reflect the context information of the peptides [9,10,11]. Transformer uses the attention mechanism to capture the association between the target word and other words in the text in order to enhance the target word’s semantic representation, but the target word itself will consist of the semantics of the main parts, leading to the information contained in each word after encoding being more inclined to the meaning of the word itself [12,13], which could not obtain high quality context features.

With the continuous development of natural language processing (NLP), the researchers used a new NLP technology-BERT to capture the context information. BERT adopts mask language model (MLM) to strengthen the dependence on context and uses output vector as the semantic representation of the whole sentence, thus integrating the semantic information of each word in the text more “fairly” and solve the problem of self-bias [14]. It has been successfully applied to protein post-translational modification site prediction [15], peptide recognition [7,16] and gene sequence study [11,17,18]. Therefore, BERT can effectively fuse the information of each amino acid to capture the context information of peptides and enhance the prediction performance of peptide detectability.

There are numerous factors that affect peptide detectability, including sequence information, physicochemical properties [19,20,21,22], context information derived from sequences, etc. [8,9]. Tang et al. [1] encoded the sequence derived information of peptides and input the feedforward neural network to predict the detection ability of peptides. Li et al. [19] considered the 292-dimensional physicochemical properties, such as peptide length, constructed artificial neural networks to predict peptide detectability. Guruceaga et al. [21] screened the 106-dimensional physicochemical properties of peptides, such as molecular weight and theoretical isoelectric point, constructed random forest classifier (RF) for peptide detection. Serrano et al. [8] extracted context information derived from sequences and input it to a convolutional neural network for feature learning, which improved the prediction accuracy of peptide detectivity. In the above studies, sequence information, physicochemical properties, and context information derived from sequences were used to predict peptide detectivity. However, these methods only considered information at a certain level of peptide, resulting in the simplification of the constructed feature space and the absence of some information. Zhang et al. [23] considered sequence information, sequence derived pseudo-amino acid composition information, and physicochemical properties, using LightGBM to predict non-classical secreted proteins. Xu et al. [24] used evolutionary information, the physicochemical properties of proteins, and sequence derived K-spaced amino acid pairs information to predict lysine succinylation sites by support vector machine (SVM), indicating that multivariate representation could make up for the unicity of feature space and effectively improve the accuracy of site prediction. The sequence information, physicochemical properties, and sequence derived information of peptides represent peptides from different perspectives. Considering three kinds of information simultaneously to predict peptide detectability can help the model to learn diversified feature representations, obtain richer feature information of peptide, and produce more reliable prediction results. Therefore, this paper intends to use sequence information, physicochemical properties, and context information derived from sequences to construct a multivariate feature space, enrich the embedded feature representation of peptides, and help the model to better predict peptide detectability.

In classification problems, heterogeneity among information is an important data characteristic of information itself [25]. Fully considering the differences between different information can help the model fully explore the characteristics of each information representation, and learn the high-quality features of specific information. The sequence information, physicochemical properties and context information describe the peptide from different perspectives. After encoding the information, the data dimension and embedding vector represented by each information are completely different, and there is great heterogeneity among the three types of information. Gao et al. [26] collected the physicochemical properties and peptide digestibility, and linearly linear fuse these information to obtain 588-dimensional vectors, used RF to predict peptide detectability. Yu et al. [27] considered the physicochemical properties and sequence information, used same CapsNet network to learn the features of the two types of information, respectively, and thus achieved the prediction of peptide detectability. These methods considered the information of different types of peptides and effectively predicted peptide detectability. However, when linear fusion or the same network is used to process different categories of information, the heterogeneity between different categories of information is not considered, which leads to the captured features losing the unique properties of the corresponding category information [28,29] and then affecting the prediction performance of the model. Ensemble deep learning strategy “specialized” a single kind of specific information [30]. Different deep learning technologies are adopted to learn the representation of specific information, which can capture the high-quality features of specific information, solve the heterogeneity problem among different information, and finally ensemble the classification results of multiple independent deep learning models. It has good fault tolerance and reliability. At present, ensemble deep learning has been successfully applied in biological sequence studies [31,32,33,34], genome analysis [35], medication adherence [36] and other fields, providing strong support for the prediction of peptide detectability.

Based on this, we propose PD-BertEDL, an ensemble deep learning method to predict peptide detectability. We introduced the dynamic bidirectional word embedding model BERT to capture the context information of peptides, and combined the context information with the sequence information and physicochemical information of peptides to construct multivariate feature space of peptides. Aiming at three types of different information of peptides, the ensemble deep learning strategy was introduced, and different deep learning methods were selected to capture the high-quality features of specific information: convolutional neural network (CNN) and bidirectional long short-term memory network (BiLSTM) were used to extract the local and global features of sequence information; CNN and BiLSTM were used to study the physicochemical characteristics of amino acids; and BiLSTM was used to learn the context characteristics of the peptides and to describe the global information of the sequence better. Then, the average fusion strategy was utilized to integrate the model prediction results based on the three types of features as the final prediction result to achieve the prediction of peptide detectability.

## 2. Results and Discussion

### 2.1. Performance of Different Encoding Schemes Based on Context Information

In order to verify whether the BERT vector adopted can capture the context information of peptide more effectively, we compared BERT with other two methods: word2vec and transformer. Among them, word2vec used CBOW algorithm, transformer used multi-head self-attention mechanism (head nums = 16), BERT used BERT-mini (which had 11.3 million parameters with 4 transformer layers and 256 hidden embedding sizes). For the three methods, Dense layer was used for classification after coding, and Sn, Sp, ACC, MCC, AUC, and AUPR values were calculated. The results are shown in Table 1.

From Table 1, compared with transformer and word2vec, the model achieves the highest value in all indicators when BERT is used to encode context information. Specifically, the ACC, MCC, AUC, and AUPR values for the BERT method are 68.95%, 37.89%, 74.99%, and 70.54%, respectively, improving by 9.48%, 18.93%, 11.50%, and 9.35% over the word2vec method, respectively. The word2vec method takes several words around the target word in the form of sliding window as samples for training. After training, the word vector of the corresponding word is obtained by looking up table [10]. No matter the amino acid context in the sequence, the word vector in the table is fixed. Therefore, the word2vec method does not take the context information of the sequence into account. In the pre-training process, BERT can calculate the association between words at each position and other words in the sequence, which can fully consider the relationship between amino acids and amino acids in the peptide sequence and can capture the context information of the peptide more effectively. Compared with transformer, the ACC, MCC, AUC and AUPR values of the BERT method are increased by 1.88%, 3.72%, 2.23%, and 2.55%, respectively. Since the information contained in each word after transformer encoding is more biased to its own meaning, the context information is not comprehensive. However, after multi-layer transformer of BERT, each output vector fairly and effectively integrates the information of each word in the entire sequence, which can better describe the global information of the peptide sequence and better predict the performance that can be obtained.

### 2.2. Ablation Experiment

#### 2.2.1. Feature Combination Ablation Experiment

In this paper, we combined sequence information, physicochemical information, and context information of peptides to characterize peptides. To verify whether the multivariate feature space constructed using three kinds of peptide information could help to predict peptide detectability, we conducted validation on the *Homo sapiens* training set based on single and multiple features of peptides, and the experimental results are shown in Table 2.

Different columns in Table 2 represent different single features or feature combinations. Among them, Feature A means sequence feature, Feature B means physical and chemical feature, and Feature C means context feature, columns 2–4 represent single feature information. Columns 5–7 indicate that integrating the two kinds of feature using average fusion strategy. Last column indicates that integrate the sequence feature, physical and chemical feature and context feature of peptide using average fusion strategy, which is the feature combination method adopted in this paper. Table 2 records the ACC, MCC, AUC and AUPR values obtained under different feature combinations. As can show in Table 2, when the three features of peptide were integrated, the ACC, MCC, AUC and AUPR values were 82.58%, 66.52%, 88.32%, and 84.01%, respectively, which were better than other conditions. Compared with the case of a single feature, the feature combination method in this paper increases by 1.43%, 1.41%, and 2.16% in ACC, respectively. Compared with the two feature combinations, the feature combinations in this paper are improved by 0.98%, 1.44%, and 1.38% in ACC, respectively. This is because the case of a single feature and two feature combinations only considered part of the feature of the peptide, resulting in the absence of some features, which cannot describe the peptide information more comprehensively. Peptide detectability is influenced by many factors. Considering the sequence information, physicochemical information, and context information derived from sequences meanwhile, the model can learn diversified feature representations, obtain rich feature information of peptides, and significantly improve the prediction performance of the model [37].

#### 2.2.2. Performance of Ensemble Deep Learning

In order to verify whether the ensemble deep learning strategy of PD-BertEDL method can effectively solve the heterogeneity problem among different information and improve the prediction performance of peptide detectability, we designed and implemented three methods: Linear Fusion [26], Same Network [27], and Hybrid [38,39,40], respectively. Among them, Linear Fusion linearly spliced the three kinds of peptide information used in this paper, input the spliced vector into CNN+BiLSTM network to extract features and used softmax to classify. The Same network used the same CNN+BiLSTM network to extract the features of the three kinds of peptide information and used the ensemble strategy based on average fusion to achieve peptide detectability prediction. Hybrid used CNN+BiLSTM, CNN+BiLSTM, BiLSTM to extract the sequence information, physicochemical information and features of context information derived from the sequence, respectively. The three obtained features were fused by matrix concatenation, and BiLSTM was used to learn the fused features. Finally, softmax was used for classification. In order to guarantee the fairness of the experiment, the coding work based on different peptide information and the parameter setting of the model in the above three methods is consistent with the proposed method. On the *Homo sapiens* dataset, we trained the four models, respectively, and evaluated the models with independent test set. The experimental results are shown in Table 3.

From Table 3, compared with Linear Fusion, Same Network, and Hybrid methods, the proposed method PD-BertEDL achieves the highest ACC, MCC, AUC, and AUPR values. Compared with the Linear Fusion method, PD-BertEDL method can extract features of different information, avoid the interference between different information, and effectively improve the quality of features. Compared with Same Network method, PD-BertEDL method uses different deep learning models to extract the features of three kinds of peptide information and learns the specific features of different information. Meanwhile, the ACC, MCC, AUC, and AUPR values are improved by 0.78%, 1.66%, 0.55% and 0.41%, respectively. Compared with Hybrid method, PD-BertEDL method adopts the ensemble method based on average fusion to integrate models based on three kinds of peptide information, give play to the advantages of each model, solve the constraints of different model training, and improve the overall prediction performance of the model. For Hybrid and PD-BertEDL, the difference is only in the final classification method. The MCC value of PD-BertEDL increases by 2.64% compared with Hybrid, and the MCC value can be used to measure the classification quality of a binary classifier [41]. This indicates that in the prediction of peptide detectability, after using different models to automatically obtain corresponding high-level features from specific information, ensemble classification method is more helpful to improve the prediction performance of peptide detectability than mixed model classification method. In summary, this paper adopts different deep learning methods to extract features of different information and adopts the ensemble strategy based on average fusion to design the ensemble deep learning method architecture reasonably and effectively, which can better predict peptide detectability.

### 2.3. Performance of Machine Learning and Deep Learning Methods

**Figure 1:**
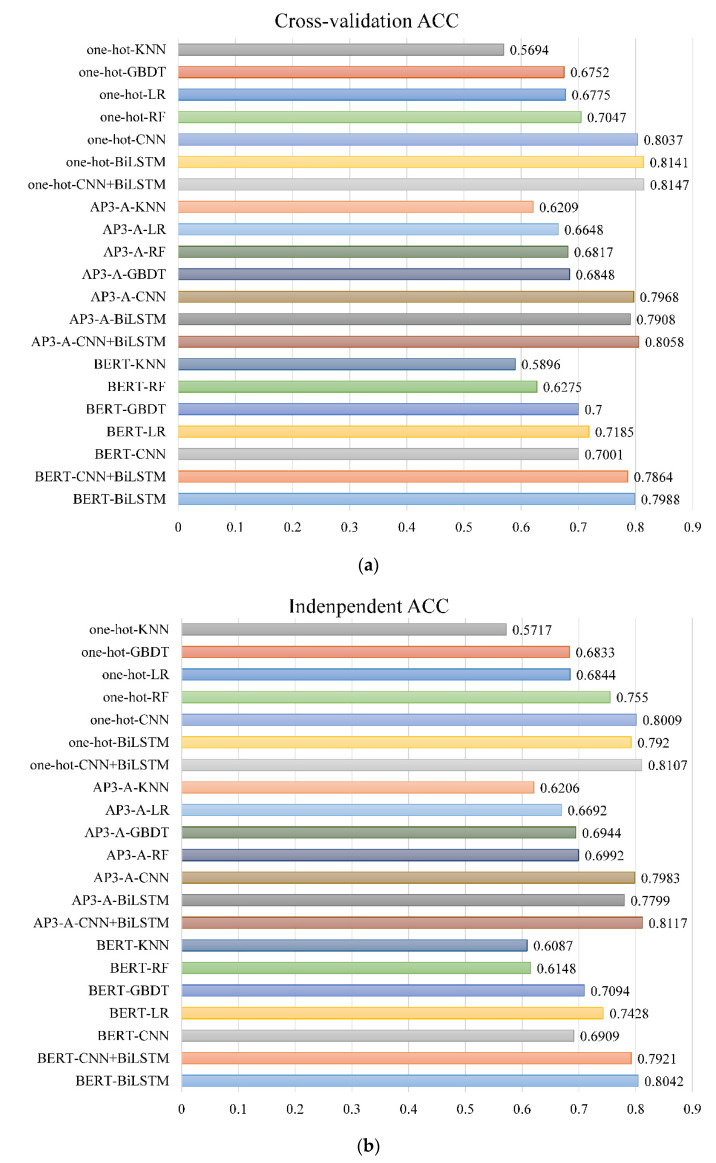
Performance of different methods on the *Homo sapiens* dataset: (**a**) 5-fold cross-validation mean results of 21 methods; (**b**) Independent test results of 21 methods.

In this paper, we used sequence information, physicochemical information, and context information derived from sequences as three kinds of information representation of peptides. In order to explore effective learning methods based on these three kinds of information, exploit the unique properties of each information fully, and capture high-quality features, we adopted four classical machine learning algorithms: K-Nearest Neighbor (KNN), Logistic Regression (LR), RF and Gradient Boosting Decision Tree (GBDT) and two deep learning algorithms: CNN and BiLSTM, and the combination model of two algorithms. Training was conducted on the *Homo sapiens* dataset, respectively, and cross-validation mean results and independent test results were obtained, as shown in Figure 1 (see Tables S3 and S4 and Figures S1–S4 of Supplementary Materials for specific experimental results).

As can be seen from Figure 1, in the 5-fold cross-validation and independent test experiments, the three deep learning methods achieve higher accuracy compared with the traditional machine learning algorithms. For the sequence information encoded of one-hot coding, when CNN+BiLSTM combined model was used to learn features, the ACC value of cross validation reaches 81.47%, which is 1.10% higher than that of CNN model only and 0.06% higher than that of BiLSTM model only. For the physicochemical information of AP3-A coding, the ACC value of cross validation reached 80.58% when CNN+BiLSTM combined model was used to learn features, which was 0.90% and 1.50% higher than that of CNN model and BiLSTM model alone, respectively. This indicates that the combination model of CNN and BiLSTM can simultaneously extract the local information of amino acid residues and the order dependent relationship between different residues and obtain higher quality sequence features and physicochemical characteristics. However, we found that for the context information of peptides, only BiLSTM was used to learn features, which achieved the optimal result; its cross-validated ACC value was 9.87% higher than that of CNN model alone and 0.12% higher than that of CNN+BiLSTM model. This is because BERT uses the above and below of the target word to capture the context information of the sequence and fully reflect the global information of the sequence. While CNN uses the sliding window to form a specific scale matrix to capture features, which will weaken the context information extracted by BERT and even lead to losing part of the information in the process of noise reduction, reducing the prediction performance of the model [42]. BiLSTM can well capture the bidirectional semantic dependence, strengthen the context information extracted by BERT, to improve the prediction accuracy of peptide detectivity.

### 2.4. Evaluation of PD-BertEDL Prediction Ability

To evaluate the prediction performance of the proposed method PD-BertEDL, we compared the prediction performance of the proposed method PD-BertEDL with other existing methods based on the independent test set. In this part of the experiment, we chose four comparison methods: DNN [22], CapsNet [27], DeepMS [8], and PepFormer [9]. Among them, DNN method only used the single information representation of the peptide to predict peptide detectability. The CapsNet method used peptide sequence information and physicochemical information to describe the peptide, and used the same network to extract features from different information to achieve the prediction of peptide detectability. DeepMS and PepFormer are meaningful attempts of deep learning and NLP technology in predicting peptide detectability: DeepMS regarded peptide sequence as a sentence and used two-layer CNN to extract the context features of peptides encoded by word2vec for further prediction; PepFormer used transformer to capture the context features of peptides and used BiGRU to predict peptide detectability, which opens the idea for the application of deep learning and NLP technology in predicting peptide detectability. In order to ensure the fairness of the experiment, training is carried out on the training set of *Homo sapiens*, and the model is evaluated on the independent test set. The experimental results are as shown in Table 4.

From Table 4, we know that compared with other predictors, the Sn, ACC, and MCC values of the proposed PD-BertEDL method are 92.38%, 82.58%, and 66.52%, respectively, but the Sp value is relatively low. Sn and Sp are antagonistic, and Sn value represents the percentage of data predicted as positive cases in all positive cases [41]. In protein identification analysis and peptide detection, it is important to know which theory digested peptides can be identified. This means that higher Sn value is more helpful in predicting peptide detectability [26]. Through analysis, it was found that the proposed method PD-BertEDL achieves the highest Sn value, which indicates that our method obtains better prediction performance due to its ability to accurately predict positive samples. In addition, the ACC value of PD-BertEDL is enhanced by 13.88%, 1.27%, 10.74%, and 0.41% compared with DNN, CapsNet, DeepMS, and PepFormer, respectively. Through analysis, the DNN method uses only the physicochemical information of peptides, while PD-BertEDL method uses the sequence information, physicochemical information, and context information of peptides to enrich the characteristic representation of peptides and solve the problem that the information representation is simple due to the use of physicochemical information only. Compared with the CapsNet method, PD-BertEDL adopts different deep learning methods for feature learning of three different kinds of peptide information, which can extract the important features of each information more effectively. At the same time, PD-BertEDL combines with the ensemble strategy to effectively integrate the prediction results of three kinds of feature information and solve the heterogeneity problem among different information and enhances peptide detectability. Compared with the deep learning and NLP methods (DeepMS and PepFormer), PD-BertEDL introduces BERT to capture the context information of peptide more fully, and combines BiLSTM to capture the bidirectional semantic dependence well, strengthens the context information extracted by BERT, and effectively improves the overall prediction accuracy of the model.

**Figure 2:**
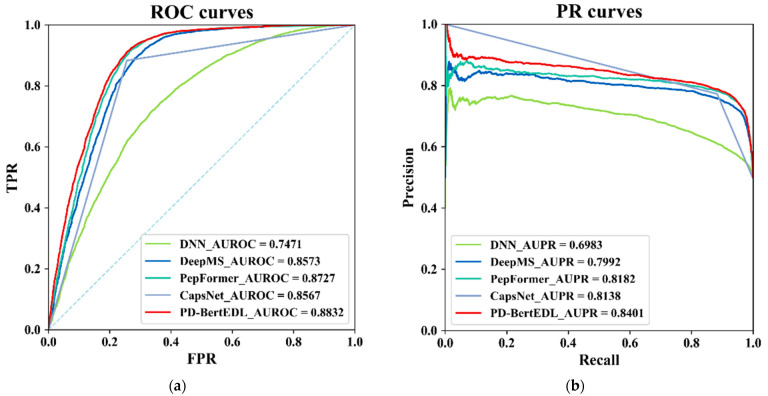
The results of PD-BertEDL and other predictors: (**a**) AUROC values; (**b**) AUPR values.

Since the ROC curve and PR curve can more intuitively compare the performance of each predictor, the ROC curve and PR curve of PD-BertEDL and other predictors on the independent test set of *Homo sapiens* were plotted in this paper, as shown in Figure 2.

The AUC and AUPR of PD-BertEDL are 88.32% and 84.01%. Compared with DNN, CapsNet, DeepMS, and PepFormer, the AUC (AUPR) value of the PD-BertEDL method increased by 13.61% (14.18%), 2.65% (2.63%), 2.59% (4.09%), and 1.05% (2.19%), respectively. In conclusion, these results demonstrate that our method retains a better predictive power than the existing methods for the peptide detectability prediction.

In order to prove the stability of PD-BertEDL, we conducted 5-fold cross-validation experiment on the Homo sapiens dataset for DNN, CapsNet, DeepMS, Pepformer, and PD-BertEDL, and recorded the results in Table 5.

It can be seen from Table 5 that the standard deviations of Sn, Sp, ACC, MCC, AUC, and AUPR of PD-BertEDL method are 0.0134, 0.0113, 0.0042, 0.0137, 0.0049, and 0.0051, respectively. Based on the results of 5-fold cross-validation, we calculated the variance of Sn, Sp, ACC, MCC, AUC, and AUPR, which were 2.24 × 10^−4^, 1.59 × 10^−4^, 2.17 × 10^−5^, 2.32 × 10^−4^, 3.00 × 10^−5^, and 3.19 × 10^−5^, respectively. (The variance of indicators for all models in Table S5). The results show that the model can learn the characteristics of peptide sequence well when processing each fold of samples, then effectively realize the prediction of peptide detectability, and it has a certain stability.

In order to further verify the generalization ability of the proposed method PD-BertEDL, the DNN, CapsNet, DeepMS, and PepFormer methods were trained on the *Musculus* dataset and independently tested and verified. The prediction results are shown in Table 6.

As can be seen from Table 6, on the *Musculus* dataset, the proposed method PD-BertEDL still achieved the optimal results on a whole. This indicates that PD-BertEDL has good generalization performance and is suitable for data of different species. In addition, we find that the three methods DNN, DeepMS, and PepFormer reach the same conclusion as the proposed method. However, prediction accuracy obtained by these three methods are lower than that of the PD-BertEDL method. For the CapsNet method, the accuracy is significantly lower than other methods. The underlying reason is that the learning ability of this model is weak and it cannot fully learn the unique characteristics of data of different species [9]. Further confirmed, even from the perspective of different species, our method is more effective for predicting peptide detectability but also proves that the method has a higher complexity and a stronger ability to learn; it can learn the unique features of different species data; and it provides a useful reference for the prediction of peptide detectability in other species.

### 2.5. t-SNE Visualization

**Figure 3:**
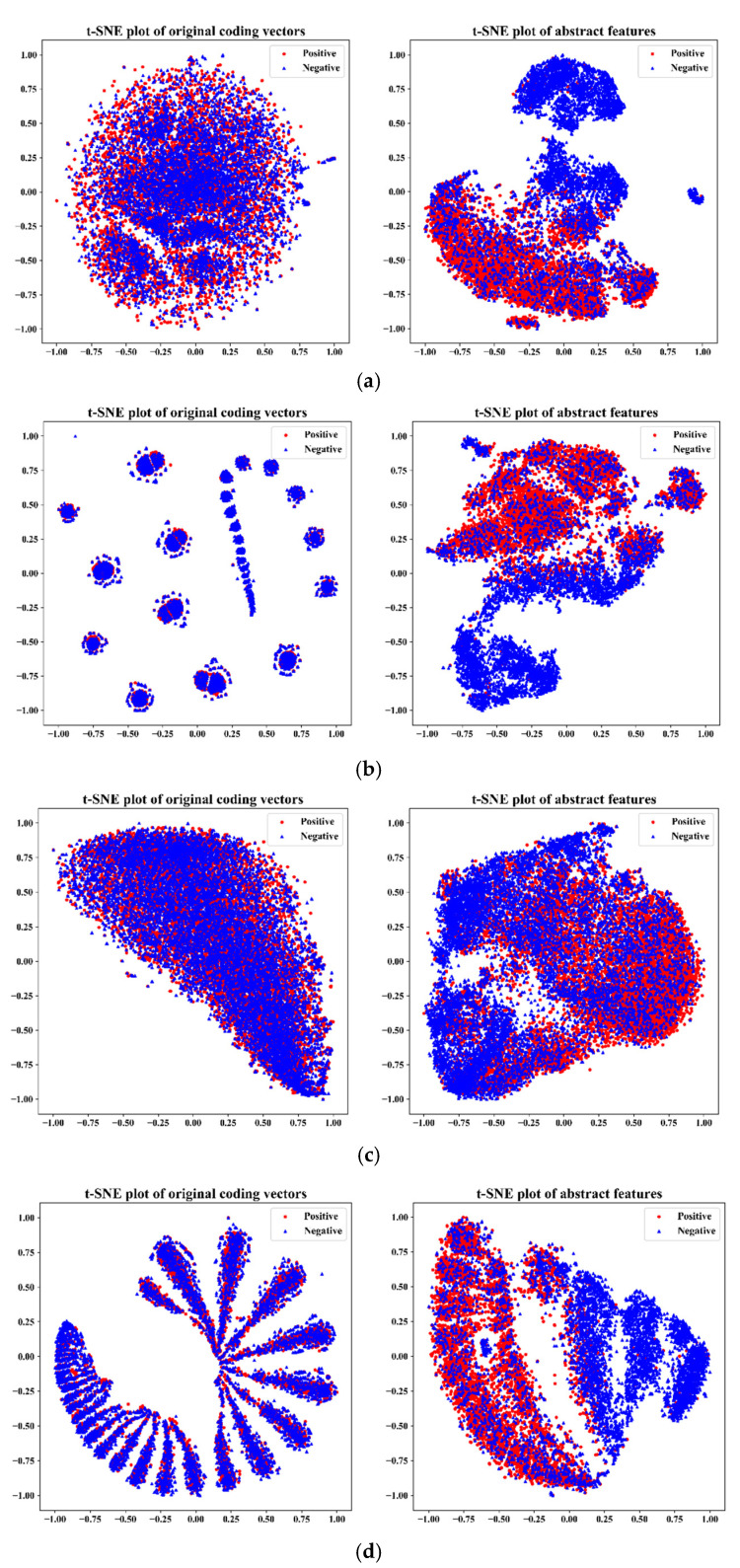
t-SNE visualization results on the independent test set of Homo sapiens: (**a**) t-SNE of sequence information; (**b**) t-SNE of physicochemical information; (**c**) t-SNE of context information; (**d**) t-SNE of three information.

In this paper, we combined sequence information, physicochemical information, and context information derived from sequences to characterize peptides for the prediction of peptide detectability. To verify that all three kinds of information contribute to the prediction of peptide detectability, we performed visual verification using t-SNE on the independent test set of *Homo sapiens*, based on two modes of single information and three combinations of information. The initial coding vectors of all samples and high-level abstract features extracted from models based on different information are projected into a two-dimensional space, and the space is scaled to the interval [−1, 1]. The results are shown in Figure 3.

For each information of peptide, after feature learning by corresponding models, each model generates a better high-level feature discrimination representation. Compared with the single information to distinguish whether a peptide can be detected or not, the multivariate information of the integrated peptide shows better discrimination. Therefore, the proposed model can learn the abstract deep representation of peptides from the sequence information, physicochemical information, and context information, enhance the distinguishing ability of features, and help to predict peptide detectability.

## 3. Materials and Methods

**Figure 4:**
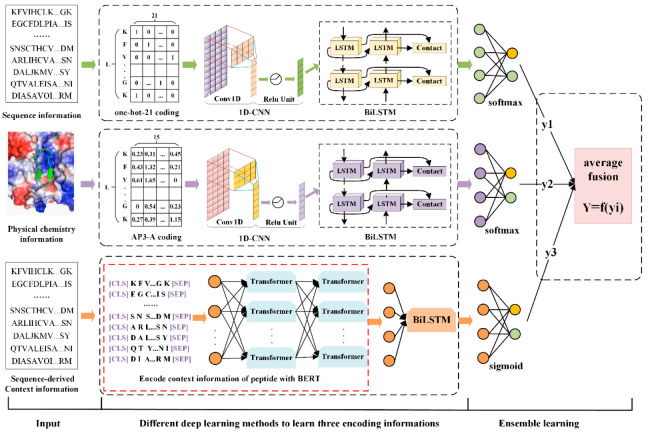
The architecture of PD-BertEDL.

Prediction of peptide detectability can be abstracted as a binary classification problem. The collected sequence can be classified as: the peptides detected by MS and the non-detected peptides [8]. We propose an ensemble deep learning method to investigate the underlying mechanisms of peptide detectability. The architecture of PD-BertEDL is shown in Figure 4.

### 3.1. Dataset Collection and Preprocessing

The dataset applied for training and testing originates from the GPMDB database [43], involving mass spectrometry data and detection frequencies for proteins identified by mass spectrometry [27]. From Cheng et al. [9], we obtained data of two species: *Homo sapiens* and *Musculus*. In order to avoid the deviation of the model due to the high sequence homology, we used the CD-HIT tool [44] with the threshold of 0.9 to delete sequences containing non-standard amino acids and redundant information. In addition, we calculated the peptide sequence lengths of the two datasets, and the maximum sequence lengths of *Homo sapiens* and *Musculus* after CD-HIT were 46 and 63, respectively. For the integrity of the sequence, we set the maximum length to the sequence length L. For sequence fragments containing fewer than L amino acids, we filled them with pseudo amino acids (represented by ‘X’). After that, we randomly selected 20% of them as an independent test set. The remaining peptide sequences were used as a training set. The statistical information of *Homo sapiens* and *Musculus* dataset is shown in Table 7.

### 3.2. Information Encoding

#### 3.2.1. Sequence Information

One-of-21 encoding was used to encode sequence information of the peptide, which is a discrete representation of value 1 at the index corresponding to the amino acid in the peptide and 0 at all other positions [41]. For example, the one-of-21 encoding of a sequence fragment ‘KFVICHLKGK’ is [[001000000000][000001000000]……[000000010000]]. Thus, for a sequence fragment with length L, L × 21-dimensional vector representation would be obtained after one-hot-21 coding.

#### 3.2.2. Physicochemical Information

Gao et al. [26] selected 15 amino acid indices from AAindex [45] as the physicochemical properties of peptides, including 10 structural indices, positive charge, 2 energy indices, hydrophobicity indices and amino acid composition (Table S1 of Supplementary Materials). In this part, the 15 amino acid indices were used to encode the physicochemical properties of the peptide, which is hereby referred to as AP3-A for the convenience of the following description. For motifs of length L, we obtained corresponding values according to the correspondence between amino acids contained in them and Table S2. For example, the corresponding physicochemical properties vector representation of sequence fragment ‘KFVICHLKGK’ is [[4.358,1.7,0.61…1,6.31], [4.663,1,0.8…0, 7.09], ……, [4.224,1.8,0.66…1,4.32]]. Thus, for a sequence fragment with length L, L × 15-dimensional vector representation would be obtained, which can represent physicochemical information.

#### 3.2.3. Sequence-derived Context Information

**Figure 5:**
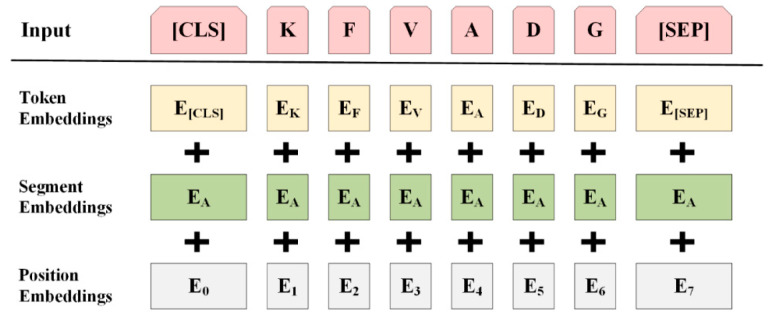
BERT coding representation.

BERT jointly adjusts the context of the text through the self-attention mechanism in all coding layers to obtain the deep bidirectional representation [46]. In the BERT model, the input vector of each word consists of three embeddings: token embedding, segment embedding and position embedding. Position embedding can represent the absolute position information of each word in a sentence [47]. A visualization of the word embedding structure is shown in Figure 5.

In order to fully capture the context information of the peptide, we regarded the peptide sequence as a sentence, and took k amino acids as a group, which was called “word” [47]. Since the parameter scale of BERT model is up to 100 million, it is very demanding for the experimental environment to use our own corpus to build a word list and retrain the BERT model. In addition, in the study of classification prediction using BERT, some research proved that the optimal results could be obtained when k = 1 was used [15,16]. Therefore, k was set to 1, and the peptide sequence of length L was divided from beginning to end to obtain L “words”. In this paper, the token sequence is an array composed of a single amino acid in the sequence. The token sequence is input into the BERT model to obtain the embedding vector representation of each amino acid. Given a sequence P = ($P_{1}$, $P_{2}$,…, $P_{L}$) ($P_{i}$ refers to each amino acid), the tokenizer transforms the sequence P and obtains the mapping ids table of the sequence, where the first CLS is the sentence vector and the last SEP is the spacer. For sequence P, we can obtain token embeddings (1, L, 256), segment embeddings (1, L, 256), and position embeddings (1, L, 256) after three layers of coding representation. A synthetic representation of size (1, L, 256) is obtained using equations (1) and fed into the BERT model.

(1) $$\mathrm{Embedding}={\mathrm{embedding}}_{\mathrm{token}}+{\mathrm{embedding}}_{\mathrm{segment}}+{\mathrm{embedding}}_{\mathrm{position}}$$

Finally, we extracted intermediate features using a pre-trained language model (Bert-mini) adopted by Qiao et al. [15], used output vector of the last layer as the context information of sequence P and used it to predict peptide detectability.

### 3.3. The PD-BertEDL Architecture

#### 3.3.1. Ensemble Deep Learning 

Ensemble deep learning strategy combines ensemble learning and deep learning technology, and can “specialized” a single feature set [30], using different deep learning methods can learn the representation of a single feature set. The ensemble deep learning strategy integrates the learning and classification results of multiple deep learning models based on a single feature set, to solve the problem of heterogeneous data combination and realize the multi-faceted abstraction of data [23]. This strategy is currently widely used to predict different biological data, such as anti-tuberculate peptides [33], antimicrobial peptides [34], recognition of non-classical secreted proteins [23], DNA recombination points [35], etc., and has been shown to help improve prediction performance and model generalization.

Considering the heterogeneity among different information, we adopted different methods to extract the features of the three kinds of peptide information, and integrated the prediction results of the model based on the three features, thus learn its unique properties from the specific information and solve the heterogeneity problem among different categories of information. We used four models with different machine learning characteristics (LR, RF, KNN, GBDT [48]) and three deep learning models (CNN, BiLSTM, CNN and BiLSTM combination model) to learn the three kinds of peptide information and predict peptide detectability. Then, we obtained the prediction results of seven models based on each kind of peptide information. Corresponding to the sequence information of peptide, the optimal model (with the highest accuracy) was selected from the seven models according to the prediction accuracy of the model as the prediction model based on the sequence information of the peptide. Similarly, corresponding to the physicochemical information of peptides and the context information derived from sequences, the optimal prediction model based on the physicochemical information of peptides and the context information derived from sequences can be obtained according to the above screening methods. Finally, predicted output probable values of the three optimal prediction models were equal-weighted averaged with average fusion strategy to achieve ensemble prediction. After training and screening, the overall prediction result Y of the ensemble model based on CNN + BiLSTM, CNN + BiLSTM and BiLSTM was obtained, as showed in Equation (2).

(2) $$Y=f\left(y_{i}\right)=\frac{1}{3}\overset{3}{\underset{i=1}{\sum }}y_{i}$$

where $y_{i}$ is the predicted probability value based on the ith information of the peptide, respectively. In this way, we effectively make use of the characteristics of each information without being affected by the number and dimension of features encoded by different information, thus enhance the performance and generalization of the model [23].

#### 3.3.2. Convolutional Neural Networks

CNN is used to extract sequence information and local features of physicochemical information of peptides. CNN carries out convolution operations through convolution kernels with shared parameters to extract local spatial information, while different convolution kernels extract different local information, and the combination of these information constitutes spatial feature, which is more efficient than fully connected deep networks. In this part, the basic CNN unit is a one-dimensional convolutional network (1D-CNN), which contains a convolution layer and a relu layer. The unit is added in a nested way to adjust the number of convolutional layers in the network. The convolution layer extracts the hidden features in the original data by summing the dot product between the convolution kernel matrix and the input data matrix. After the convolution operation, the relu activation function is used to remove the negative information and retain the useful information for classification.

Taking motifs of length L as an example, the feature extraction process of 1D-CNN is shown in Equations (3)–(5).

(3) $$X^{\prime }=f_{conv}\left(I\right)$$

(4) $$X_{i,j}^{’}=\overset{F}{\underset{F=1}{\sum }}\overset{R}{\underset{r=1}{\sum }}W_{F,r}^{i}\cdot I_{F,r}^{j}+b_{i}$$

(5) $$X^{\left(CNN\right)}=f_{relu}\left(X^{\prime }\right)=relu\left(X^{\prime }\right)=\max\left(0,X^{\prime }\right)$$

Here, $I\in {\mathbb{R}}^{F\times L}$ is the coding vector of the input layer, and $F\in \left\{21,15\right\}$ is the coding length of the sequence information and physicochemical information of the peptide. $X_{i,j}^{\prime }$ is the characteristic representation of the ith convolution kernel sliding to the jth amino acid, $i\in \left\{1,\ldots ,N_{filter}\right\}$ ($N_{filter}$ is the number of convolution kernels), $j\in \left(1,\ldots ,L\right)$ (R is the size of the convolution kernel, and S is the sliding step. $W\in {\mathbb{R}}^{N_{filter}\times F\times R}$ is the weight matrix, and b is the bias term. $X^{\left(CNN\right)}\in {\mathbb{R}}^{N_{filter}\times L}$ is the output of 1D-CNN. This process is repeated in a nested way to construct multi-layer CNN, so as to capture deeper feature information.

#### 3.3.3. Bi-Directional Long Short-Term Memory Network

**Figure 6:**
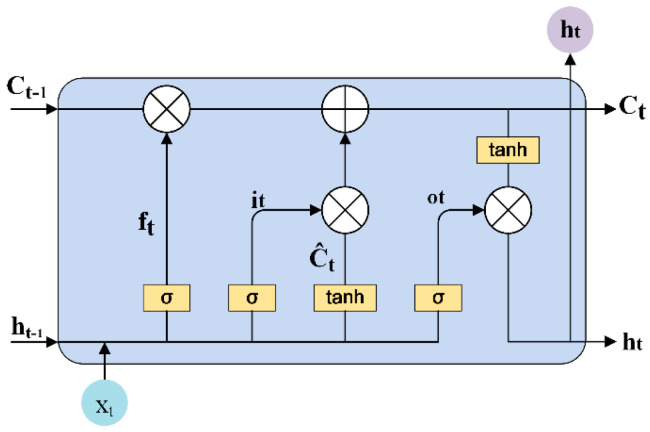
LSTM unit structure.

Considering the sequential correlation between different amino acids, BiLSTM is used to capture the interdependence information and global information among amino acids in peptide sequences, so as to enhance the information flow in the process of peptide sequence feature learning and to improve the discriminative ability of the network. BiLSTM is composed of LSTM units, and the structure of a single LSTM unit is shown in Figure 6.

After the LSTM unit receives the input data, the first step was to decide which information to keep through the “forgetting gate” and the output of the last moment. The second step was divided into two parts. Firstly, generating new information through the “input gate”; Second, new information was added through the tanh layer to update the current unit state. The third step was to get the output of LSTM unit through “output gate” and tanh layer. The calculation process of LSTM element at time step t  is shown in Equations (6)–(10).

(6) $$f_{t}=\sigma \left(W_{xf}x_{t}+W_{hf}h_{t-1}+b_{f}\right)$$

(7) $$i_{t}=\sigma \left(W_{xi}x_{t}+W_{hi}h_{t-1}+b_{i}\right)$$

(8) $$C_{t}=f_{t}C_{t-1}+i_{t}\tanh\left(W_{xC}x_{t}+W_{hC}h_{t-1}+b_{C}\right)$$

(9) $$o_{t}=\sigma \left(W_{xo}x_{t}+W_{ho}h_{t-1}+b_{o}\right)$$

(10) $$h_{t}=o_{t}\tanh\left(C_{t}\right)$$

$f_{t},\;\;i_{t},\;\;C_{t},\;\;o_{t}$ and $h_{t}$ represent forgotten gate, input gate, unit state, output gate and hidden state, respectively. $x_{t}$ represents the LSTM unit input at time step t; W and b represent the weight matrix and the bias term, respectively. Therefore, the LSTM unit adjusts the internal information flow through the gating mechanism and controls the historical information through the “forgetting gate”, to ensure that the network learns the dependence relationship between amino acid residues. BiLSTM network has outputs from two directions, which are connected in series.

### 3.4. Evaluation Metrics

Several statistical measures were considered to evaluate the performance of the proposed model and other predictors. They were sensitivity (Sn), specificity (Sp), accuracy (ACC), and Matthew’s correlation coefficient (MCC). The definitions are as follows.

(11) $$Sn=\frac{TP}{TP+FN}$$

(12) $$Sp=\frac{TN}{TN+FP}\;$$

(13) $$ACC=\frac{TP+TN}{TP+TN+FP+FN}$$

(14) $$MCC=\frac{TP\times TN-FP\times FN}{\sqrt{\left(TP+FP\right)\times \left(TP+FN\right)\times \left(TN+FN\right)\times \left(TN+FP\right)}}$$

where TP, TN, FP, and FN represent true positives, true negatives, false positives, and false negatives, respectively. In addition, we also used the area under the receiver operating characteristic (ROC) curve (AUC) and the area under the precision recall rate (PR) curve (AUPR) to further access the overall performance of the model.

## 4. Conclusions

In this paper, we proposed an ensemble deep learning method, PD-BertEDL, to achieve effective prediction of peptide detectability. We used the sequence information, physicochemical information, and context information derived from the sequence of the peptide, and input the three kinds of information into the corresponding deep learning model after encoding. In view of these three different kinds of information, CNN and BiLSTM were used to extract the peptide sequence information and the characteristics of physicochemical information. BiLSTM was used to capture the long-distance dependence information of the peptide sequence and the deep features of the context information of the peptide. Finally, we ensemble model prediction results based on a single feature representation to improve prediction performance and model generalization ability. The results of a k-fold cross-validation experiment and an independent testing experiment suggest that our method has potential to be a useful tool for the prediction of peptide detectability.

Although our method achieves promising performance in peptide detectability prediction, there is still some room for improvement. In the future, we will try to reduce the memory consumption of the model. At the same time, we will consider developing an online platform aimed at providing an effective means of predicting peptide detectability.

## Figures and Tables

**Table 1:** Performance of three encoding methods on the independent set of *Homo sapiens*.

Method	Sn (%)	Sp (%)	ACC (%)	MCC (%)	AUC (%)	AUPR (%)
BERT	69.79	68.09	68.95	37.89	74.99	70.54
transformer	68.08	66.09	67.07	34.17	72.76	67.99
word2vec	53.49	65.33	59.47	18.96	63.49	61.19

**Table 2:** Performance of single feature or multiple feature ensemble.

Feature A	√			√	√		√
Feature B		√		√		√	√
Feature C			√		√	√	√
ACC(%)	81.15	81.17	80.42	81.60	81.14	81.20	82.58
MCC(%)	62.64	63.97	61.12	64.73	64.45	64.35	66.52
AUC(%)	87.07	86.57	87.07	87.58	87.59	87.62	88.32
AUPR(%)	82.11	81.16	82.69	82.70	83.05	83.06	84.01

**Table 3:** The results of PD-BertEDL and other integration methods.

Model	ACC (%)	MCC (%)	AUC (%)	AUPR (%)
Linear fusion	81.61	65.38	88.12	83.71
Same network	81.80	64.86	87.77	83.60
Hybrid	81.02	63.88	87.18	82.26
PD-BertEDL(our)	82.58	66.52	88.32	84.01

**Table 4:** The results of PD-BertEDL and other predictors on the independent test set of *Homo sapiens*.

Model	Sn (%)	Sp (%)	ACC (%)	MCC (%)
DNN [22]	75.59	61.93	68.70	37.86
CapsNet [27]	88.32	74.43	81.31	63.34
DeepMS [8]	89.02	71.84	80.35	61.72
PepFormer [9]	81.56	79.61	82.17	61.17
PD-BertEDL(our)	92.38	72.96	82.58	66.52

**Table 5:** The results of 5-fold cross-validation with different models on *Homo sapiens* dataset.

Model	Sn (%)	Sp (%)	ACC (%)	MCC (%)	AUC (%)	AUPR (%)
DNN [22]	73.92 ± 1.71	62.10 ± 1.70	67.96 ± 0.14	36.28 ± 0.32	73.77 ± 0.42	68.88 ± 0.62
CapsNet [27]	89.98 ± 0.71	73.02 ± 1.11	81.50 ± 0.48	63.94 ± 0.93	85.97 ± 0.33	81.50 ± 0.50
DeepMS [8]	90.79 ± 1.03	68.86 ± 0.38	79.73 ± 0.38	61.08 ± 0.70	85.25 ± 0.28	79.25 ± 0.39
PepFormer [9]	82.85 ± 2.13	76.20 ± 3.36	81.96 ± 0.32	59.03 ± 3.68	87.25 ± 0.31	82.36 ± 0.79
PD-BertEDL (our)	90.25 ± 1.34	71.76 ± 1.13	82.13 ± 0.42	65.10 ± 1.37	88.35 ± 0.49	84.29 ± 0.51

**Table 6:** The results of PD-BertEDL and other predictors on the independent test set of *Musculus*.

Model	Sn (%)	Sp (%)	ACC (%)	MCC (%)	AUC (%)	AUPR (%)
DNN [22]	58.34	65.29	62.05	23.67	67.34	60.39
CapsNet [27]	35.37	82.38	60.43	20.22	64.64	58.88
DeepMS [8]	72.06	66.35	69.01	38.34	75.54	66.62
PepFormer [9]	73.25	73.77	73.88	46.94	80.78	72.20
PD-BertEDL(our)	85.29	65.53	74.76	51.41	81.21	72.99

**Table 7:** Dataset information of *Homo sapiens* and *Musculus*, where the threshold of CD-HIT is 0.9.

	Dataset Type	Positive Sample	Negative Sample
*Homo sapiens*	Training	22,404	22,813
	Independent test	5601	5703
*Musculus*	Training	7320	8356
	Independent test	1829	2089

## Data Availability

Not applicable.

## Supplementary Materials

The following are available online at: https://www.mdpi.com/article/10.3390/ijms232012385/s1.
