# Supplementary material for: PD-BertEDL: An Ensemble Deep Learning Method Using BERT and Multivariate Representation to Predict Peptide Detectability
Source: Int J Mol Sci. 2022 Oct 16;23(20):12385. doi: 10.3390/ijms232012385 (PMC9604182; doi:10.3390/ijms232012385)
Supplement: Supplementary file 1 [file ijms-23-12385-s001.zip › ijms-1922171-supplementary.pdf]

PD-BertEDL: An Ensemble Deep Learning Method Using BERT and Multivariate Representation to Predict Peptide Detectability

Huiqing Wang[1,*], Juan Wang[1], Zhipeng Feng[1], Ying Li[1] and Hong Zhao[1]

[1] College of Information and Computer, Taiyuan University of Technology, Taiyuan 030024, China
* Correspondence: wanghuiqing@tyut.edu.cn.

Supporting Tables

Table S1. Amino acid properties details of 15 physicochemical information.

category	Amino acid properties
structure	0-alpha-CH chemical shifts
	1-Helix termination parameter at posision j-2, j-1, j
	2-Optimized propensity to form reverse turn
	3-Normalized frequency of alpha-helix in all-alpha class All-alpha
	4-Correlation coefficient in regression analysis
	5-Weights for alpha-helix at the window position of -1
	6-Average relative fractional occurrence in ER(i-1)
	7-Relative preference value at N1
	8-Normalized frequency of isolated helix
	9-Normalized frequency of zeta R
Hydrophobicity	Hydrophobicity coefficient in RP-HPLC, C18 with 0.1%TFA/2-PrOH/MeCN/H2O
energy	0-Activation Gibbs energy of unfolding
	1-Slopes tripeptide FDPB PARSE neutral
charge	Positive charge
AAC	AA composition of EXT2 of single-spanning proteins

Table S2. Amino acid properties details of 15 physicochemical information value.

Amino acid	str0	str1	str2	str3	str4	str5	str6	str7	str8	str9
A	4.349	1.2	1.34	1.08	0.687	0.34	0.99	1.2	0.946	0.328
C	4.686	1	1.07	1.22	0.263	-0.18	2.32	0.8	0.481	0
D	4.765	0.7	3.32	0.86	0.632	0.06	1.18	0.8	1.311	3.379
E	4.295	0.7	2.2	1.09	0.669	0.2	1.36	2.2	0.698	0
F	4.663	1	0.8	0.96	0.577	0.15	1.25	0.5	0.963	1.336
G	3.972	0.8	2.07	0.85	0.67	-0.88	1.4	0.3	0.36	0.5
H	4.63	1.2	1.27	1.02	0.594	-0.09	1.06	0.7	2.168	1.204
I	4.224	0.8	0.66	0.98	0.564	-0.03	0.81	0.9	1.283	2.078
K	4.358	1.7	0.61	1.01	0.407	-0.11	0.91	0.6	1.203	0.835
L	4.385	1	0.54	1.04	0.541	0.2	1.26	0.9	1.192	0.414
M	4.513	1	0.7	1.11	0.328	0.43	1	0.3	0	0.982
N	4.755	1.2	2.49	1.05	0.489	-0.33	1.15	0.7	0.432	1.498
P	4.471	1	2.12	0.91	0.6	-0.81	0	2.6	2.093	0.415
Q	4.373	1	1.49	0.95	0.527	0.01	1.52	0.7	1.615	0
R	4.396	1.7	0.95	0.93	0.59	0.22	1.19	0.7	1.128	2.088
S	4.498	1.5	0.94	0.95	0.692	-0.35	1.5	0.7	0.523	1.089
T	4.346	1	1.09	1.15	0.713	-0.37	1.18	0.8	1.961	1.732
V	4.184	0.8	1.32	1.03	0.529	0.13	1.01	1.1	0.409	0.946
W	4.702	1	-4.65	1.17	0.632	0.07	1.33	2.1	1.925	1.781
Y	4.604	1	-0.17	0.8	0.495	-0.31	1.09	1.8	0.802	0
	**Hydrophobicity**	**Energy0**	**Energy1**	**charge**	**AAC**					
A	-2.34	-0.729	18.56	0	5.04					
C	5.03	-0.408	17.84	0	2.2					
D	-0.48	-0.545	17.94	0	5.26					
E	1.3	-0.532	17.97	0	6.07					
F	2.57	-0.454	17.95	0	3.72					
G	-1.06	-0.86	18.57	0	7.09					
H	-3	-0.519	18.64	1	2.99					
I	7.26	-0.361	19.21	0	4.32					
K	1.56	-0.508	18.36	1	6.31					
L	1.09	-0.462	19.01	0	9.88					
M	0.62	-0.518	18.49	0	1.85					
N	2.81	-0.597	18.24	0	5.94					
P	-0.15	NA	18.77	0	6.22					
Q	0.16	-0.492	18.51	0	4.5					
R	1.6	-0.535	0	1	3.73					
S	1.93	-0.278	18.06	0	8.05					
T	0.19	-0.367	17.71	0	5.2					
V	2.06	-0.323	18.98	0	6.19					
W	3.59	-0.455	16.87	0	2.1					
Y	-2.58	-0.439	18.23	0	3.32					

Table S3. Cross-validation mean results and independent test results of 21 classifiers on the Mus.culus dataset.

		Cross-validation	Independent test
**Descriptor**	**Classifier[a]**	**mean_Acc±std_Acc**	**Acc**
One-hot	kNN	0.5202±0.0052	0.5368
	LR	0.5996±0.0097	0.6154
	RF	0.5731±0.0037	0.5914
	GBDT	0.6093±0.0163	0.6212
	CNN	0.7129±0.0112	0.7112
	BiLSTM	0.7407±0.0041	0.7430
	CNN+BiLSTM	**0.7434±0.0046**	**0.7502**
AP3-A	kNN	0.5377±0.0090	0.5500
	LR	0.5960±0.0050	0.6041
	RF	0.5593±0.0129	0.5773
	GBDT	0.6199±0.0153	0.6276
	CNN	0.7190±0.0106	0.7242
	BiLSTM	0.7162±0.0079	0.7142
	CNN+BiLSTM	**0.7336±0.0084**	**0.7331**
BERT-mini	kNN	0.5472±0.0072	0.5521
	LR	0.6050±0.0052	0.6307
	RF	0.5478±0.0081	0.5516
	GBDT	0.6059±0.0072	0.6217
	CNN	0.6214±0.0104	0.6195
	BiLSTM	**0.7037±0.0081**	**0.7055**
	CNN+BiLSTM	0.6197±0.0134	0.6252

a kNN: k-nearest neighbor, LR: logistic regression, RF: random forest, GBDT: Gradient Boosting Decision Tree. The best results for each descriptor are highlighted in bold.

Table S4. Cross-validation mean results and independent test results of 21 classifiers on the Homo.spaines dataset.

		Cross-validation	Independent test
**Descriptor**	**Classifier[a]**	**mean_Acc±std_Acc**	**Acc**
One-hot	kNN	0.5694±0.0046	0.5717
	LR	0.6775±0.0097	0.6844
	RF	0.7047±0.0072	0.7550
	GBDT	0.6752±0.0061	0.6833
	CNN	0.8037±0.0020	0.8009
	BiLSTM	0.8141±0.0041	0.7920
	CNN+BiLSTM	**0.8147±0.0032**	**0.8107**
AP3-A	kNN	0.6209±0.0115	0.6206
	LR	0.6648±0.0111	0.6692
	RF	0.6817±0.0113	0.6992
	GBDT	0.6848±0.0092	0.6944
	CNN	0.7968±0.0039	0.7983
	BiLSTM	0.7908±0.0067	0.7799
	CNN+BiLSTM	**0.8058±0.0078**	**0.8117**
BERT-mini	kNN	0.5896±0.0111	0.6087
	LR	0.7185±0.0050	0.7428
	RF	0.6275±0.0149	0.6148
	GBDT	0.7000±0.0104	0.7094
	CNN	0.7001±0.0243	0.6909
	BiLSTM	**0.7988**±0.0068	**0.8042**
	CNN+BiLSTM	0.7864±0.0098	0.7921

a kNN: k-nearest neighbor, LR: logistic regression, RF: random forest, GBDT: Gradient Boosting Decision Tree. The best results for each descriptor are highlighted in bold.

$\times 10^{-5}$

Table S5. The variance of indicators with different models.

	**Sn**	**Sp**	**ACC**	**MCC**	**AUC**	**AUPR**
DNN	3.65×10^{-4}	3.59×10^{-4}	2.56×10^{-6}	1.26×10^{-5}	2.25×10^{-5}	4.77×10^{-5}
CapsNet	6.30×10^{-5}	1.53×10^{-4}	2.93×10^{-5}	1.07×10^{-4}	1.40×10^{-5}	3.08×10^{-5}
DeepMS	1.33×10^{-4}	2.17×10^{-4}	1.81×10^{-5}	6.13×10^{-5}	9.47×10^{-6}	1.87×10^{-5}
PepFormer	5.66×10^{-4}	1.41×10^{-3}	1.29×10^{-5}	1.69×10^{-3}	3.76×10^{-5}	7.76×10^{-5}
PD-BertEDL	2.24×10^{-4}	1.59×10^{-4}	2.17×10^{-5}	2.32×10^{-4}	3.00×10^{-5}	3.19×10^{-5}

Supporting Figures

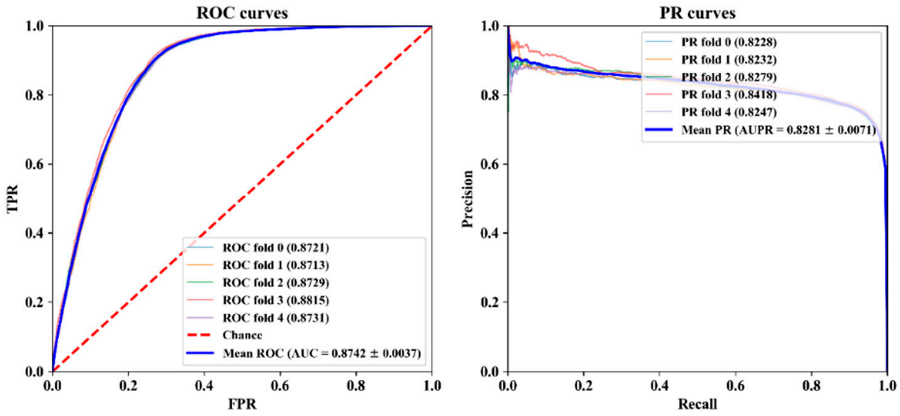

(a) AUROC and AUPR curve of sequence information-CNN+BiLSTM.

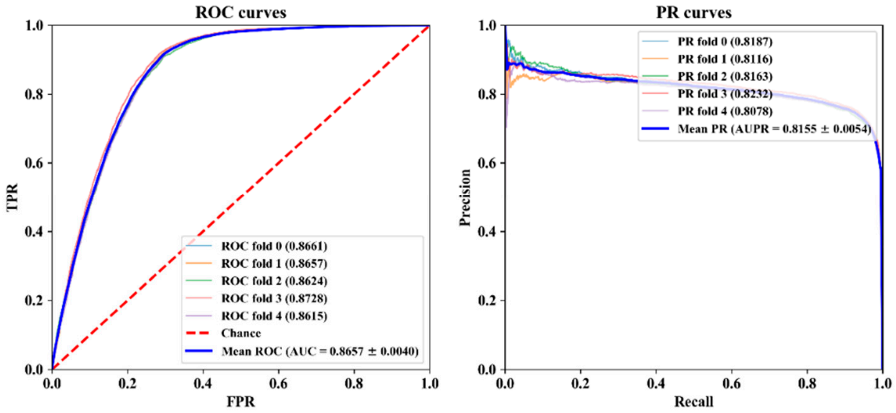

(b) AUROC and AUPR curve of physicochemical information-CNN+BiLSTM.

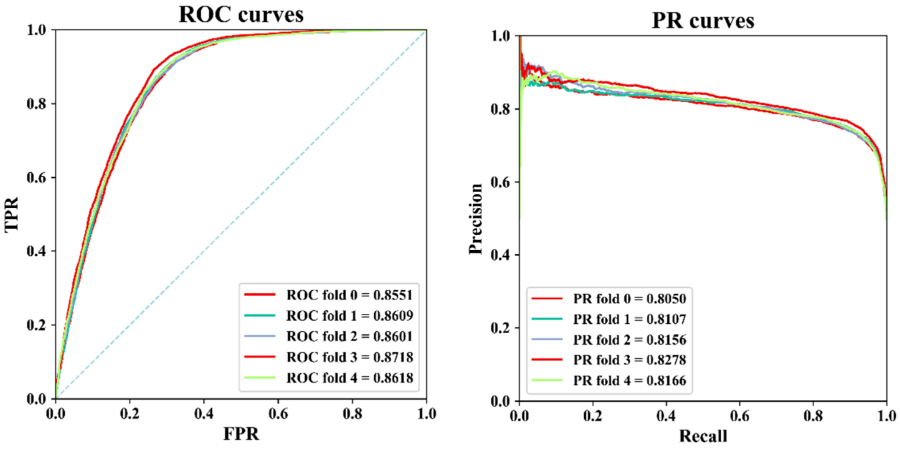

(c) AUROC and AUPR curve of context information-BiLSTM.

Figure S1. Cross-validation results of DL classifiers with three feature descriptors on the Homo.sapiens dataset.

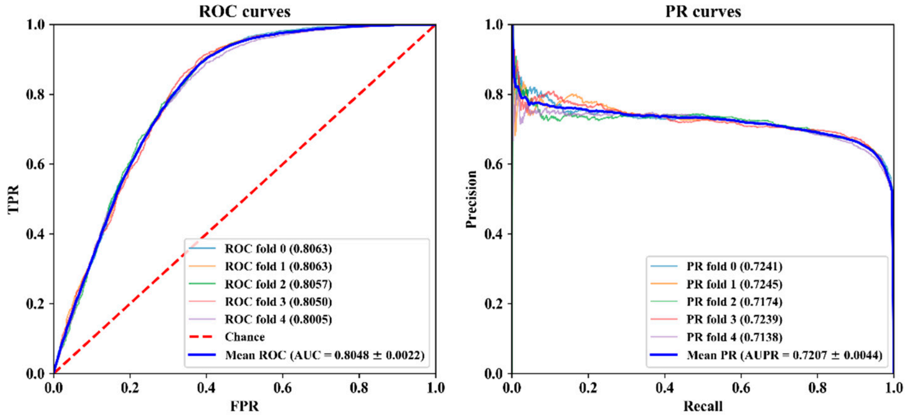

(a) AUROC and AUPR curve of sequence information-CNN+BiLSTM.

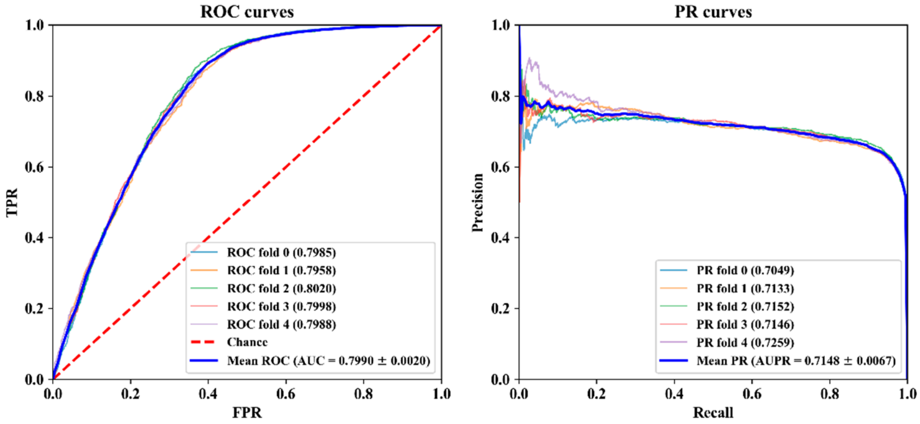

(b) AUROC and AUPR curve of physicochemical information-CNN+BiLSTM.

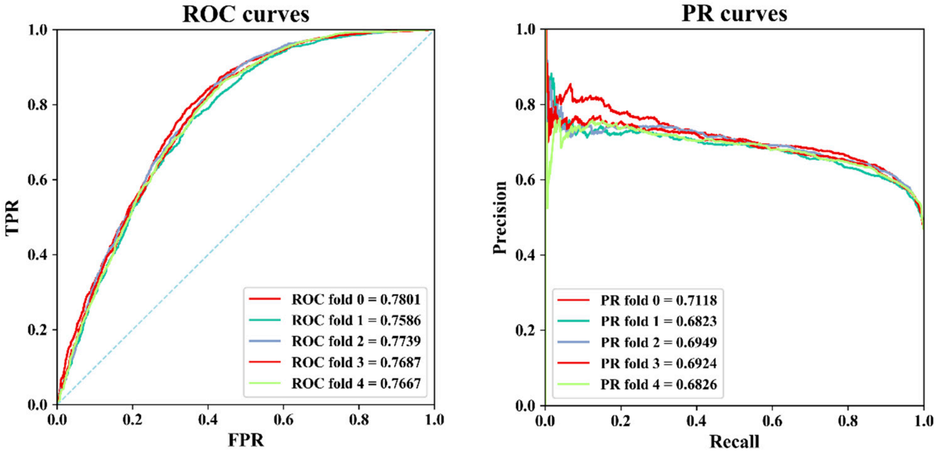

(c) AUROC and AUPR curve of context information-BiLSTM.

Figure S2. Cross-validation results of DL classifiers with three feature descriptors on the Mus.culus dataset.

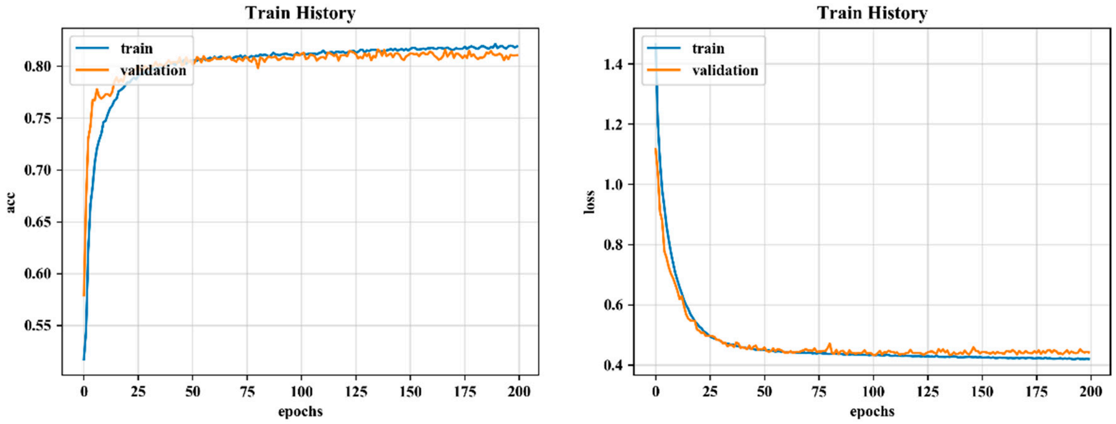

(a) Accuracy and loss curve of sequence information-CNN+BiLSTM.

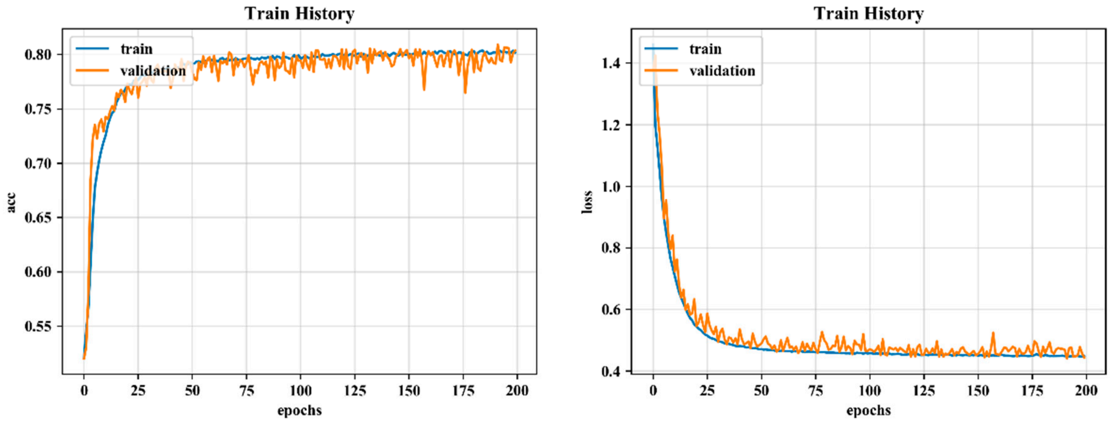

(b) Accuracy and loss curve of physicochemical information-CNN+BiLSTM.

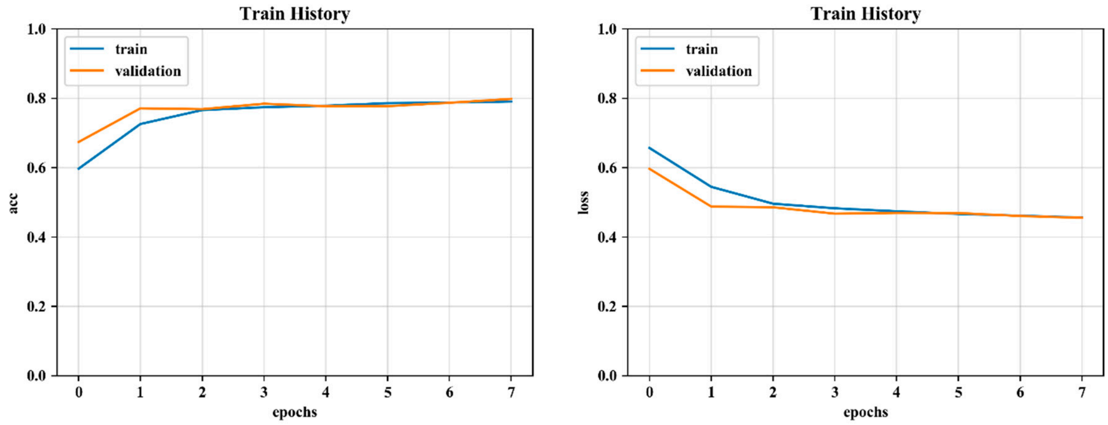

(c) Accuracy and loss curve of context information-BiLSTM.

Figure S3. Accuracy and loss of the feature1-3 on the Homo.sapiens dataset during training.

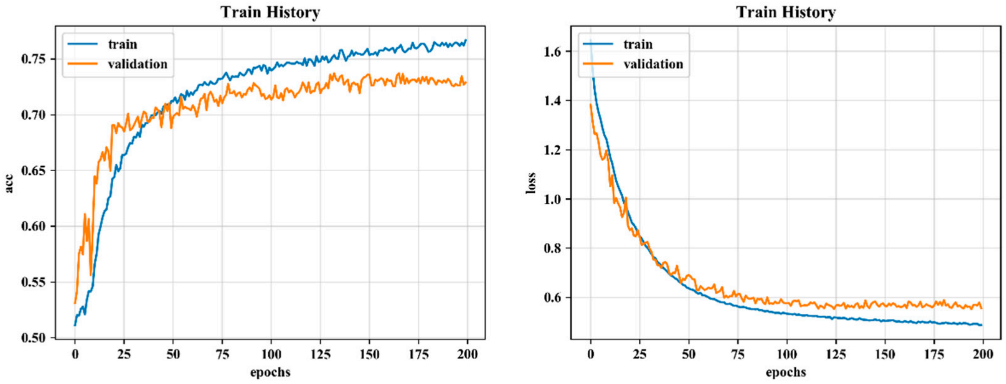

(a) Accuracy and loss curve of sequence information-CNN+BiLSTM.

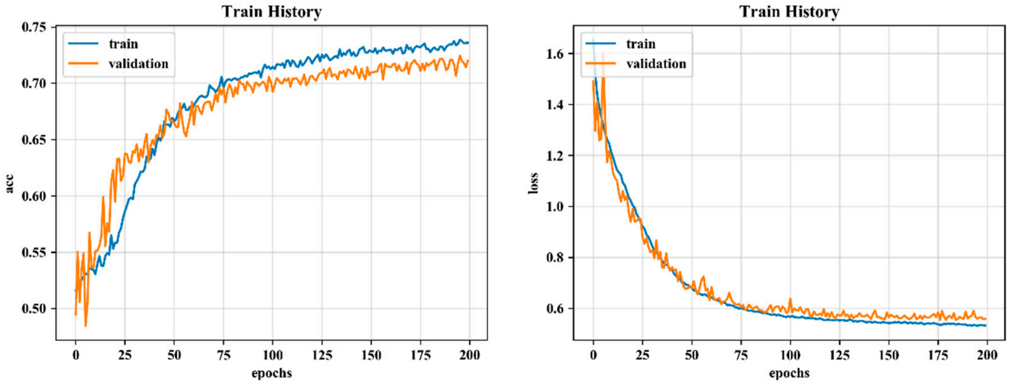

(b) Accuracy and loss curve of physicochemical information-CNN+BiLSTM.

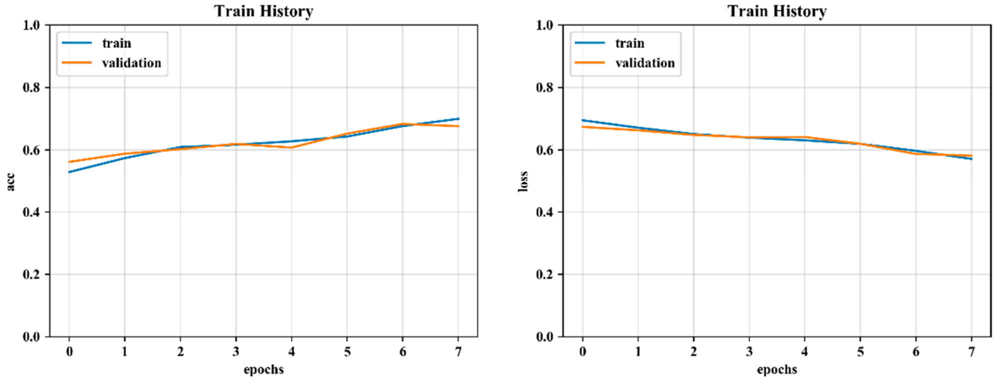

(c) Accuracy and loss curve of context information-BiLSTM.

Figure S4. Accuracy and loss of the feature1-3 on the Mus.culus dataset during training.
